# Effect of Foot Orthoses on Midfoot Pain and the Volume of Bone Marrow Lesions in the Midfoot: A Randomized Mechanism of Action Study

**DOI:** 10.1002/acr.25648

**Published:** 2025-12-08

**Authors:** Jill Halstead, Anne‐Maree Keenan, Philip G. Conaghan, Dennis McGonagle, Anthony C. Redmond

**Affiliations:** ^1^ Leeds Institute of Rheumatic and Musculoskeletal Medicine University of Leeds Leeds United Kingdom; ^2^ Leeds Community Healthcare NHS Trust Leeds United Kingdom; ^3^ School of Healthcare University of Leeds Leeds United Kingdom; ^4^ NIHR Leeds Biomedical Research Centre Leeds Teaching Hospitals Trust Leeds United Kingdom; ^5^ Versus Arthritis Experimental Osteoarthritis Treatment Centre Leeds United Kingdom; ^6^ Centre for Sports, Exercise and Osteoarthritis, Versus Arthritis, Nottingham and Leeds United Kingdom

## Abstract

**Objective:**

Foot orthoses are thought to improve pain by potentially modifying internal mechanical forces. To test this, we explored whether foot orthoses can modify patterns of bone marrow lesions (BMLs) in people with midfoot pain.

**Methods:**

Forty‐two people were recruited with midfoot pain, and magnetic resonance imaging–confirmed midfoot BMLs. Participants were randomized (2:1 ratio) to receive either pre‐formed orthoses (n = 27) or control cushioning insoles (n = 15). Outcomes included foot pain (visual analog scale [VAS]), pain and functional impairment subscales of the Manchester Foot Pain and Disability Index, and BML volume measured at baseline and 12 weeks.

**Results:**

In total 108 bones in the midfoot were identified with BMLs (mean 2.5 bones, SD 1.6). In the orthoses group, pain significantly reduced at 6 weeks (mean VAS = −14.8 mm, confidence interval [CI] −22.3 to −7.3) and 12 weeks (mean VAS = −7.1 mm, CI −15.0 to −0.9) compared to the control group at 6 weeks (mean VAS = −7.4 mm, CI −19.9 to 5.2) and 12 weeks (mean VAS = 2.8 mm, CI −9.1 to 14.7). In the orthoses group, functional impairment and pain impairment were significantly reduced at 6 weeks and to a lesser extent at 12 weeks. In the control group, only the functional impairment reduced significantly at 6 weeks. At 12 weeks, BML volume reduced more in the orthoses group (−1544.4 mm^3^, CI −3660.4 to 571.6), compared to the control group (−315.8 mm^3^, CI −1528.2 to 896.7).

**Conclusion:**

The foot orthoses group showed a greater reduction in foot pain and a greater reduction in the volume of BMLs compared with the control group.

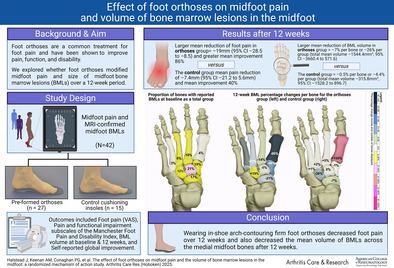

## INTRODUCTION

The prevalence of foot pain has been reported to be between 13% and 36%, and in the midfoot region, the prevalence is 19% over the age of 50 years.^1^, ^2^ For people with midfoot pain, walking impairment and disability is similar to midfoot osteoarthritis (OA).^2^, ^3^ Foot orthoses are a common treatment for foot pain and have been shown to improve pain, function, and disability in a range of soft tissue and joint disorders.^4^ Gait studies show foot orthoses can alter joint motion, change indirect joint forces, and redistribute foot pressure.^5^ The mechanism by which foot orthoses improve pain, however, is poorly understood due to the limitations of direct structural measures.

**SIGNIFICANCE & INNOVATIONS**

- This is the first clinical study to explore the impact of foot orthoses on magnetic resonance imaging–detected bone marrow lesions in people with midfoot pain.
- Wearing in‐shoe arch‐contouring firm foot orthoses decreased foot pain over 12 weeks.
- Wearing in‐shoe arch‐contouring firm foot orthoses also decreased the mean volume of bone marrow lesions across the medial midfoot bones after 12 weeks.

Indirect measures of bone stress and adaptive physiologic changes can be captured using magnetic resonance imaging (MRI), which is sensitive to fluid changes in the bone, defined as bone marrow lesions (BMLs). This nonspecific feature has been shown to reflect physiologic bone changes as well as short‐term and long‐term adaptive bone stress.^6^ Peripheral BMLs have been shown to be responsive to changes in loading and fatigue in the legs and feet,^7^, ^8^ associated with increased stress in runners^9^ and sports,^10^ miliary,^11^ and dance participants.^12^ These BMLs can be a feature of asymptomatic and symptomatic groups undergoing high levels of joint stress, in which the locations are known to be related to repetitive biomechanical activities.^13^ In people with pain, BMLs can be reflective of bone stress and underlying OA.^3^, ^14^ In people with knee pain, BML size has been shown to correlate weakly with pain, with such pain potentially related to trabecular changes, increased bone vascularity, and perivascular innervation.^14^, ^15^, ^16^ The biomechanical relationship between BML and joint and bone pain has been established in animal models of OA^17^ and in human knee^14^ and hip studies.^18^ In the foot, however, this exploration has been limited by a lack of imaging studies and difficulties in tissue sampling. Although our cross‐sectional study found the number of people with midfoot pain and bones with BMLs per person was not related,^19^ a further study of BMLs in people with midfoot pain and midfoot OA (compared to a control group) showed the size of the BMLs was related to the severity of midfoot pain.^3^

To study the biomechanical adaptation of bone stress in humans, gait studies and imaging studies have explored the effect of orthotic interventions. In the foot, wearing immobilization boots after injury has been shown to resolve BML patterns^20^, ^21^ and providing foot orthoses for military training has been shown to prevent bone stress injuries.^22^ These foot studies explored orthotic devices and their influence on bone stress in an acute injury model; however the mechanism and role of foot orthoses in a community population with established midfoot pain has not been explored.

To further understand the mechanism of action of foot orthoses, we hypothesized that BMLs at the site of midfoot pain were modifiable by wearing foot orthoses. The aim was to explore whether foot orthoses modified midfoot pain and size of midfoot BMLs over a 12‐week period.

## PATIENTS AND METHODS

This study had an experimental study design, employing a randomized method to investigate the mechanism of action of foot orthoses and was a substudy of a larger cohort examining the relationship between midfoot pain and BMLs.^19^ All eligible participants from the larger study were invited to the orthoses intervention study (International Standard Randomised Controlled Trial Number 77862746).

Participants (aged 18 years or over) were invited from the Leeds Community Musculoskeletal and Rehabilitation service (from 2008 to 2011 in Leeds, United Kingdom) with midfoot pain over three months duration and pain with weight‐bearing activity. If participants presented with bilateral pain, the most painful foot was chosen as the study foot, or if the pain was considered equal, a single foot was identified by asking the participants to initiate walking and the first step was determined to identify limb dominance.^19^

Eligible people were invited for an MRI scan on a single foot to meet the inclusion criteria. Two experienced readers (rheumatologist and radiologist) confirmed at least one BML, defined as a hyper‐intense signal (visualized on water sensitive T2‐weighted fat‐saturated or short tau inversion ratio sequences and hypo‐intense signal on T1‐weighted sequences) in a region greater than a quarter of one bone in the seven medial midfoot bones (medial cuneiform, intermediate cuneiform, lateral cuneiform, navicular, and proximal first to third metatarsals).

People with midfoot pain were not eligible if they already wore foot orthoses or presented with clinical signs of midfoot OA, defined as observed or palpable osteophytes, reduced range of motion, or identification by radiology report as having features of established midfoot OA using standard clinical foot radiographs (measured in standard anterior–posterior and oblique planes). Other exclusion criteria were foot surgery in the last 12 months and/or internal metal fixation, foot pain typical of undiagnosed or diagnosed inflammatory arthritis, neurologic pain or signs and symptoms of sensory loss and a medical history of diabetes mellitus, peripheral arterial disease, kidney disease, organ transplantation, or being fitted with a pacemaker or any other implant contra‐indicated for MRI. In accordance with the Declaration of Helsinki, ethical approval was provided by the Leeds Ethics Committee (reference: 09/H1305/10), and all participants provided informed written consent.

Randomization was conducted on a 2:1 basis favoring the active orthoses intervention over the control (sham) group; this approach was chosen to favor the intervention and explore structural and clinical outcomes. Randomization was provided by senior researcher (AR) using a random number protocol and blind (opaque) envelope allocation, which was revealed to the researcher (JH) sequentially at the baseline appointment. Assessor and participants were not blinded; however, the firm (active) versus cushioning (sham) devices were presented with equal merit to the participants to limit bias.

**Figure 1:**
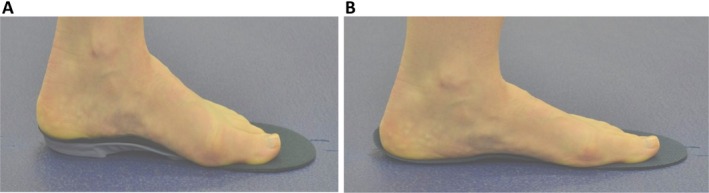
(A) Foot orthoses group—Vectorthotic. (B) Control group—cushioning sham insole.

Participants who were randomized to the intervention group received a pair of Vectorthotic foot orthoses (Healthy Step Ltd): a three‐quarter length polypropylene composite shell with rearfoot medial wedges added (either 4° or 6°) and Vectorthotic Extra full length top cover (6 mm to 14 mm variable depth of compressed polyethylene foam). The control group received the Vectorthotic uniform top cover comprised of 4‐mm compressed foam. It was expected that the cushioning sham insole may reduce load in the heel and forefoot to a small degree, whereas the Vectorthotic was chosen to increase midfoot contact force and maximum midfoot force^23^, ^24^; see Figure 1A and B and Supplementary S1 for fitting details.

The primary clinical outcome of this study was foot pain severity in the last 24 hours using a 100‐mm visual analog scale^25^, ^26^, ^27^ reported at baseline, 6 weeks, and 12 weeks and MRI‐detected BML volume, which was measured at baseline and 12 weeks. The secondary outcome included foot pain–related disability (including functional subscale and the pain subscale), measured using the Modified Manchester Foot Pain and Disability Index (MMFPDI)^28^, ^29^ and self‐reported global improvement by asking if the foot pain is the same, improved, or worse at the 6‐week and 12‐week appointment.^30^ All outcomes were collected by an unblinded assessor.

The imaging outcomes used an MRI scan at baseline and 12 weeks with a single foot scanned at both timepoints using a Siemens Magnetom Verio (3T) large bore MRI scanner (Siemens Medical Solutions). As described previously,^19^ all images were acquired using an eight‐channel foot and ankle coil, with the foot placed perpendicular to the ankle and magnetic field (β0). To minimize positional error, all images were measured manually from the scout image to ensure that the foot was perpendicular to the ankle in the sagittal plane and centered over the navicular bone at each visit with the following protocol. T2‐weighted fat‐saturated sequence parameters were as follows: repetition time (TR): 3,000 to 3,600 milliseconds, echo time (TE): 69 milliseconds, flip angle: 155° to 160°, echo train length: 8, 2 mm slices and 0.4 mm interslice gap, matrix 256 × 256 pixels, field of view (FOV) 150 × 150 mm. Short tau inversion ratio sequence parameters were as follows: TR: 4,500 milliseconds, TE: 31 milliseconds, number of excitations: 2, inversion recovery time: 200 milliseconds, flip angle: 150°, echo train length: 11, 3 mm slices and 0.6 mm interslice gap, matrix 320 × 256 pixels, FOV 150 × 150 mm in all three planes. T1‐weighted sequence parameters were spin echo high‐resolution, TR: 700 milliseconds, TE: 10 milliseconds, flip angle: 90°, 1.2 mm slices and 1.32 mm interslice gap, matrix 512 × 512 pixels, FOV 512 × 512 mm, in the sagittal plane.

### Image analysis

The measurement of bones and BMLs were conducted using anonymized MRIs to ensure the researcher was blinded to group allocation and sequence of scans. Each bone with BMLs was segmented using Analyze software version 10 (AnalyzeDirect). The borders of which were identified using a manual tracing for each cross‐sectional area per slice of bone. Once the volume of the bone was determined, the signal of normal bone was identified by manually selecting the lower and upper range of the marrow greyscale signal, which was subtracted from the bone through an automated function leaving the defined BML hyper‐intense signal (see Supplementary File 2). This software provided two outputs: a cross‐sectional slice area of the bone and a cross‐sectional area defined as BML signal. With the additional information of slice thickness and the slice gap, the volume of bone and BML per bone was calculated (Volume = ∑ Area Bone × [slice thickness + interslice gap]). The individual bone volumes and BML volumes were calculated per scan providing the outcome on a group level and bone level at baseline and follow‐up. The percentage BML reduction was also calculated between visits (baseline volume − final volume ÷ baseline volume × 100) on a group level.

The repeatability of this method was investigated in 20 participants; a total of 78 bones measured repeated in a random order over one week later. For the midfoot bones, the absolute difference (root mean squared error) was 1,338.2 mm^3^ or 5% of the mean bone volume of the total bones per participant. For BMLs, the absolute difference (root mean squared error) was 427.8 mm^3^ or 6.2% of the mean volume of each bone.

### Statistical analysis

Differences between the baseline and follow‐up scores were reported using the mean and SD, and trends were examined using a two‐way repeated measures *t*‐test (level of significance was set at α = 0.05), and 95% confidence intervals (CIs) were calculated. As this was an exploratory investigation, full inferential analysis was not proposed for this study. Descriptive analysis of demographic data and outcomes were undertaken using SPSS version 28 (IBM).

## RESULTS

**Figure 2:**
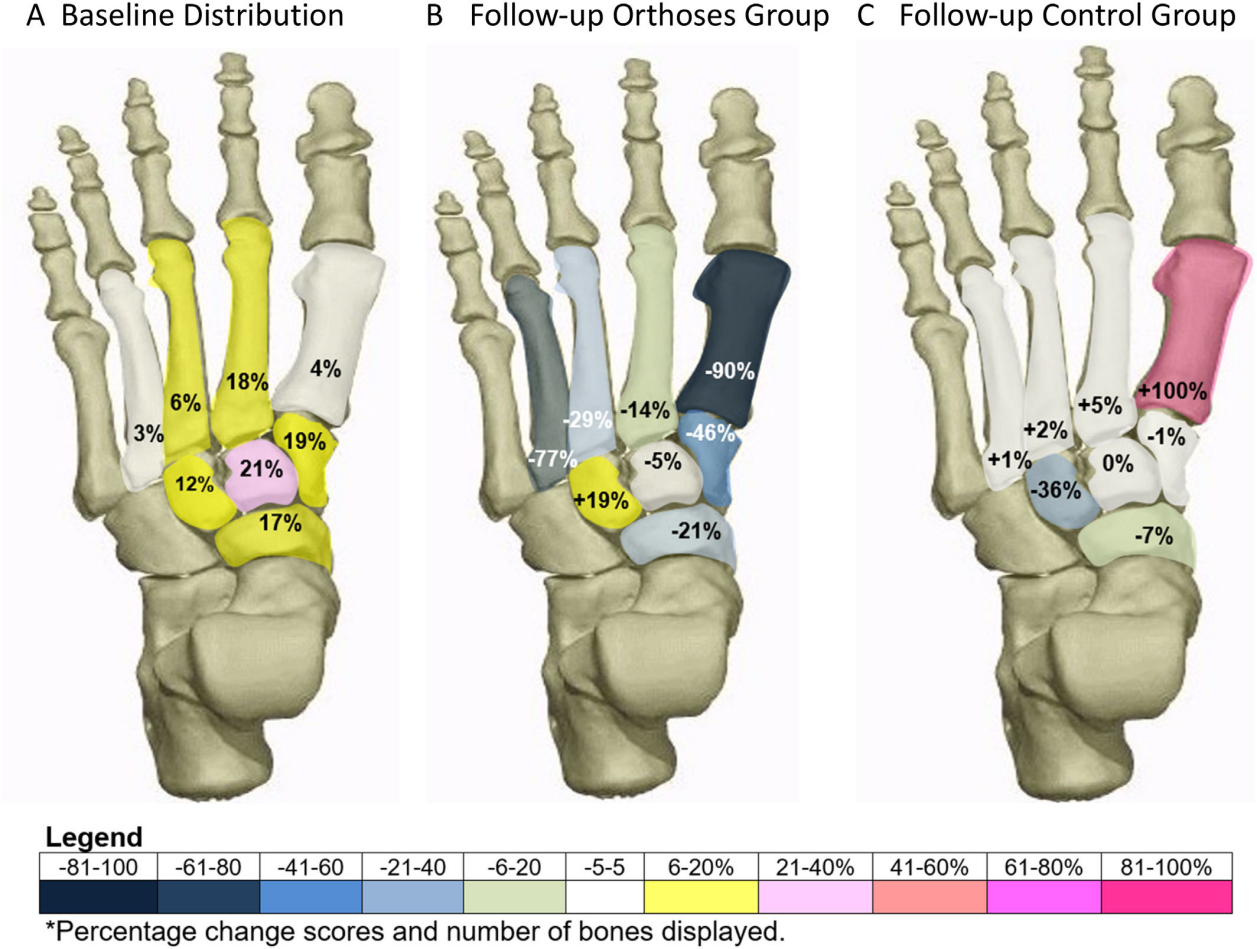
(A) shows the proportion of bones with reported BMLs at baseline as a total group. (B) and (C) show the 12‐week BML percentage changes per bone for the (B) orthoses group and (C) control group. BML, bone marrow lesion. Color figure can be viewed in the online issue, which is available at http://onlinelibrary.wiley.com/doi/10.1002/acr.25648/abstract.

Sixty‐one people with midfoot pain were invited to have a 3T MRI scan: once the scans were scored, 45 participants with mechanical midfoot pain and confirmed medial midfoot BMLs were invited to the study; 42 were included in the study. Demographics of participants are presented in Table 1: age ranged from 22 to 72 years (mean age 53 [SD 12.2] years), a high proportion were female (71%), and 43% were classified as having obesity and 36% as having overweight (mean body mass index = 30 [SD 5.1]). The most frequently reported comorbidities were OA (n = 17), obesity (n = 13), hypertension (n = 12), and asthma (n = 7), and the most frequently reported sites of other joint pain were the knee (n = 17), hand (n = 7), neck (n = 5), and hip (n = 5). Foot pain was the only site in 33% of the group, whereas 38% reported pain in one other joint, 21% reported pain in two additional joints, and 7% reported pain in three other joints. The mean number of bones with BML per participant were evenly spread, although the mean percentage of BMLs per bone was slightly higher in the control group. The location of bones (n = 108) with BMLs at baseline are shown in Figure 2A. The intermediate cuneiform was the most common bone with BMLs (21%), followed by the second metatarsal bone (19%), and around a third of the group had a pattern of two bones involved.

**Table 1:** Demographic profile and baseline characteristics of the total group and randomized orthoses and control treatment groups*

Demographics and baseline characteristics	Total group	Orthoses group	Control group
	(n = 42)	(n= 27)	(n = 15)
Age, mean (SD), y	53 (12.2)	55 (10.8)	49 (13.6)
Gender (female), n (%)	30 (71)	20 (77)	10 (63)
BMI, mean (SD)	30.0 (5.1)	29.5 (4.1)	30.6 (6.5)
Total comorbidities, mean (SD)	2.2 (1.6)	2.1 (1.5)	2.4 (1.8)
Total painful joints, mean (SD)	1.4 (1.2)	1.7 (1.2)	0.8 (0.9)
Pain (0–100 mm), mean (SD)	34.6 (17.3)	36.5 (16.1)	32.2 (19.4)
Pain related disability, mean (SD)	30.5 (6.1)	30.6 (5.8)	30.5 (6.8)
Pain impairment, mean (SD)	13.6 (3.0)	14.1 (2.7)	12.9 (3.3)
Functional impairment, mean (SD)	16.9 (4.6)	16.5 (4.7)	17.6 (4.6)
No. of bones with BMLs, mean (SD)	2.6 (1.6)	2.6 (1.5)	2.6 (1.9)
BML volume % of bone, mean (SD)	35.1 (19.5)	31.3 (19.5)	35.1 (19.8)
Study foot	26 right	15 right	11 right

* BMI, body mass index, BML, bone marrow lesion.

### Randomization

45 participants were randomized on a 2:1 ratio for treatment with foot orthoses intervention (orthoses group n = 30) or control cushioned insoles (control group n = 15). After randomization, 3 immediately dropped out of the study in the orthoses intervention group, leaving 27 participants. Randomization and the small sample size led to characteristics being unequally distributed: age was higher in the orthoses group and number of painful joints was lower in the control group (see Table 1). An additional five from the orthoses group did not complete the three‐month follow‐up (see CONSORT statement Supplementary File 3): one participant left the study due to a flare of back pain, three experienced ankle sprains due to icy weather, and one was lost to follow‐up.

### Intervention

Adherence, number of hours wearing the foot orthoses per day, was evaluated using a daily log. At the six‐week review, the mean hours per week was slightly higher in the control group (mean 34.5 [SD 16.6] hours) compared to the orthoses group (mean 32.2 [SD 13.7] hours). At the final review, adherence remained slightly higher in the control group (mean 42.3 [SD 17.0] hours) compared to the orthoses group (mean 39.5 [22.4] hours). Orthoses adherence improved over the course of the three‐month study.

### Clinical outcomes

In the orthoses group, mean midfoot pain (see Table 2) reduced in the first 6 weeks (mean −14.8 mm, CI −22.3 to −7.3 mm), and smaller mean pain reductions were noted from 6 to 12 weeks (mean −7.1, CI −15.0 to 0.9 mm). In contrast, the control group showed small mean midfoot pain reduction in the first 6 weeks (mean −7.4 mm, CI −19.9 to 5.2 mm) and a small mean increase of foot pain between 6 and 12 weeks (mean 2.8 mm, CI −9.1 to 14.7 mm).

**Table 2:** Mean foot pain (VAS) and foot pain impairment and functional impairment (MMFPDI) and self‐reported improvement at baseline and follow‐up in each group*

Outcomes	Orthoses group						Control group					
	Week 0 (n = 27), mean (SD)	Week 6 (n = 25), mean (SD)	Week 12 (n = 22), mean (SD)	Week 0 to 6, difference (CI)	Week 6 to 12, difference (CI)	Week 0 to 12,^a^ difference (CI)	Week 0 (n = 15), mean (SD)	Week 6 (n = 15), mean (SD)	Week 12 (n = 15), mean (SD)	Week 0 to 6, difference (CI)	Week 6 to 12, difference (CI)	Week 0 to 12, difference (CI)
Foot pain	35.7 (16.2)	20.5 (17.5)	14.6 (17.9)	−14.8 (−22.3 to −7.3)	−6.0 (−15.0 to 0.9)	−18.5 (−28.5 to −8.5)	32.7 (20.0)	24.3 (22.3)	24.9 (19.5)	−7.4 (−19.9 to 5.2)	2.8 (−9.1 to 14.7)	−7.8 (−21.2 to 5.6)
Foot pain impairment	14.0 (2.7)	10.6 (3.3)	10.8 (3.0)	−3.5 (−5.1 to −1.9)	0.8 (−0.3 to 1.9)	−2.7 (−4.3 to −1)	13.0 (3.4)	11.9 (2.9)	12.1 (3.2)	−0.7 (−1.1 to 2.5)	0.1 (−1.3 to 1.5)	−0.9 (−2.4 to 0.7)
Foot function impairment	16.5 (4.7)	13.6 (4.8)	13.0 (4.2)	−3.5 (−5.1 to −1.8)	−0.4 (−1.7 to 0.9)	−3.5 (−5.2 to −1.9)	17.5 (4.8)	15.2 (4.5)	14.9 (1.7)	−2.1 (−4.3 to 0.0)	−0.4 (−2.4 to 1.7)	−2.7 (−4.0 to −1.3)
Self‐reported improvement, %												
Improved	–	76	86	–	–	–	–	53	40	–	–	–
Same	–	16	9	–	–	–	–	40	47	–	–	–
Worse	–	8	5	–	–	–	–	7	13	–	–	–

* CI, 95% confidence interval; MMFPDI, Modified Manchester Foot Pain and Disability Index; VAS, visual analog scale.

^a^ Excluding 5 participants who did not complete the study in the orthoses group.

Both groups showed small reductions of functional impairment (MMFPDI) in the first 6 weeks of orthoses therapy (intervention mean −3.5, CI −5.1 to −1.8; control mean −2.1, CI −4.3 to 0.0). There was a greater mean reduction noted in the orthoses intervention group; however, the CIs were similar in both groups. At the 12‐week follow‐up, both groups showed no change in functional impairment (orthoses mean −0.4, CI −1.7 to 0.9; control mean −0.4, CI −2.4 to 1.7).

Foot pain impairment (MMFPDI) was reduced in the orthoses group in the first 6 weeks (mean −3.5, CI −5.1 to −1.9), although at 12 weeks there was minimal change (mean +0.8, CI −0.3 to 1.9). In contrast, change of foot pain impairment in the control group after 6 weeks was mean −0.7 (CI −1.1 to 2.5) versus mean +0.1 (CI −1.3 to 1.5) for 12 weeks follow‐up. These results suggest midfoot pain and foot pain impairment reduced in the first 6 weeks in the orthoses group and not in the control group. Functional impairment reduced in both groups in the first 6 weeks of intervention and then plateaued. In the self‐reported evaluation of the pain (see Table 2), 86% of participants in the orthoses group reported improvement, compared to 40% of the control group, after 12 weeks.

### Structural outcomes

After 12 weeks, the baseline and follow‐up BML patterns were explored in each group in terms of total mean measurements for bone volume, BML volume, and BML percentage per bone (see Table 3). Mean bone volumes were similar in both groups, with some increase in bone volume measurement in the control group at follow‐up due to two more bones being detected. Although both groups showed a reduction in BML volume after 12 weeks, a larger reduction was noted in the orthoses group by a mean of −1544.4 mm^3^ (CI −3660.4 to 571.6 mm^3^) with an overall percentage decrease of 25.8% and percentage reduction per bone of −6.6% (CI −14% to 0.8%). In the control group, there was a smaller mean reduction of −315.8 mm^3^ in BML volume (CI −1528.2 to 896.7 mm^3^), with an overall percentage decrease of 4.4% and a percentage reduction per bone of −0.5% (CI −6.6% to 5.8%).

**Table 3:** The mean difference between 0 and 12 weeks follow‐up BML outcomes per group*

Bone and BML measurements	Orthoses group		Difference (CI)	Control group		Difference (CI)
	Week 0^a^	Week 12	n = 22	Week 0	Week 12	n = 15
Bone volume, mean (SD) mm^3^	18,380.5 (9,828.4)	18,300.5 (9,453.4)	−79.9 (−556.7 to 396.8) *P* = 0.730	19,750.5 (15,763.3)	20,234.7 (16,269.2)	484.2 (−51.9 to 1,020.3) *P* = 0.73
BML volume, mean (SD) mm^3^	5,977.3 (5,023.0)	4,432.9 (3,239.5)	−1,544.4 (−3,660.4 to 571.6) *P* = 0.144	7,183.8 (9,125.4)	6,868.1 (8,533.3)	−315.8 (−1,528.2 to 896.7) *P* = 0.587
BML per bone, n (%)	33.2 (20.8)	26.6 (19.5)	−6.6 (−14.0 to 0.8) *P* = 0.078	35.1 (19.8)	34.6 (24.3)	−0.5 (−6.6 to 5.8) *P* = 0.892

* *P* is the probability of 2‐tailed *t*‐test, significance set at 5%. BML, bone marrow lesion, CI, 95% confidence interval.

^a^ Excluding 5 participants who did not complete the study in the orthoses group.

New BML formation was noted in both groups at 12 weeks. In the control group, four participants reported new BML formation in varying degrees of size in the navicular, medial cuneiform, intermediate cuneiform, and first metatarsal bones (5%, 6%, 11%, and 100% of bone, respectively). In the orthoses group, one participant had new BMLs in three bones: lateral (58%), medial (17%), and intermediate (41%) cuneiforms. The other three BMLs were newly formed in three intermediate cuneiforms (64%, 6%, and 6% of bone). None of the BML resolutions occurred in the same participants with new BML formation.

The BML results were further explored on bone level for the relative change in number of bones and percentage BML volume per group (see Table 4 and Figure 2B and 2C). Figure 2 presents the distribution of bones with BMLs across the midfoot at baseline, which demonstrates that the intermediate cuneiform, medial cuneiform, navicular, and second metatarsal were most affected. It also shows the percentage change of BML volume per bone per intervention and control group at 12 weeks. In the orthoses group, a total of seven bones showed complete resolution of BML, and the remaining seven of the eight BML bone sites showed some reduction, whereas in the control group, two bones with BML resolved, and five of the eight BML bone sites showed some BML reduction. In the case of the medial cuneiform and the intermediate cuneiform bones, there were increases and decreases in BML volume. In the orthoses intervention group, the mean percentage bone reductions were in the medial cuneiform, navicular, and metatarsal bones. In contrast, these bones showed very little mean change in the control group.

**Table 4:** Frequency and change of BMLs per bone site for the total group and each treatment arm: orthoses and control groups at 0 and 12 weeks follow‐up*

		Orthoses group				Control group			
Groups	Total group		Weeks 12 BML change (n = 22)			Week 0 (n = 15)	Week 12 BML change (n = 15)		
Time point (participants)	Baseline (N = 42)	Week 0 (n = 22)^a^		Bone change, n	Mean BML vol change,^b^ %			Bone change, n	Mean BML vol change, %
Total bones	108	54	53			42	44		
Bone sites, n (%)									
1st metatarsal	4 (4)	2 (4)	1 (2)	−1	−90	0 (0)	1 (2)	+1	+100
2nd metatarsal	20 (19)	10 (18)	9 (17)	−1	−15	8 (19)	8 (18)	0	+5
3rd metatarsal	6 (6)	3 (6)	3 (6)	0	−28	3 (7)	3 (7)	0	−2
4th metatarsal	3 (3)	1 (2)	1 (2)	0	−77	2 (5)	2 (5)	0	+1
Medial cuneiform	20 (19)	11 (20)	11 (20)	+1/−1	−46	6 (14)	6 (14)	+1/−1	−7
Intermediate cuneiform	23 (21)	11 (20)	11 (20)	+4/−4	−4	9 (21)	9 (20)	+1/−1	0
Lateral cuneiform	14 (12)	6 (11)	7 (13)	+1	+24	7 (17)	7 (16)	0	−36
Navicular	18 (18)	10 (18)	10 (19)	0	−25	7 (17)	8 (18)	+1	−1

* BML, bone marrow lesion; vol, volume.

^a^ Excluding 5 participants who did not complete the study in the orthose.

^b^ Mean BML change is the percentage reduction per bone.

## DISCUSSION

This study aimed to investigate the mechanism of action of foot orthoses in people with midfoot pain and walking difficulties, using clinical outcomes and MRI scans to measure the effect on BMLs as a surrogate measure of bone stress. In this novel study, midfoot pain and MRI‐detected bone signal in the midfoot were assessed before and after 12 weeks of wearing either firm foot orthoses or cushioning sham insole. The results of this exploratory study showed that average foot pain reduced in both groups, but there was a greater reduction in the orthoses intervention group, and more people reported improvement after 12 weeks. In terms of MRI‐detected bone signal, after the intervention, total mean volume of BML and total mean percentage of BML per bone was lower in the orthoses intervention group than the control group at 12 weeks. In the intervention group, the reduction of BML size and foot pain occurred over a shorter period than previously reported hospital studies, which showed foot BMLs took six to nine months to reduce^31^ and up to one year for resolution.^32^ Pain reduction in conjunction with BML reduction suggests further studies are needed to explore the role of bone stress in midfoot pain.

In the orthoses group, the BML volume bone reductions were mostly found in the medial cuneiform, navicular, and metatarsal bones. In contrast, these same bones showed very little mean change in the control group, suggesting the orthoses may influence the distribution of the mechanical stress within the medial midfoot. On an individual bone level in the orthoses group, a greater number of bones showed either resolution of BMLs or percentage BML reductions, compared to the control group. In the case of the medial cuneiform, and particularly the intermediate cuneiform, the results suggested these bones were sensitive to changes in bone stress.

Bone marrow signal detected on MRI scans is a measure of abnormal physiology^33^ and, in the foot BMLs, are associated with high repetitive stress, such as excessive training, in running, sports, and the miliary.^34^ In a clinical study of hospital MRI scans in people with foot and ankle pain, BMLs were primarily associated with trauma (44%), OA (35%), and primary BML (6%), followed by (<5%) infection, ischemia, and neoplasm.^35^ In this community study, people were recruited with an average duration of 10 months foot pain and a pattern of weight‐bearing foot pain that was not related to trauma, and people with clinical and radiographic signs of OA were excluded. The baseline MRI characteristics of people with midfoot pain, as we have previously published, show that the number of bones with BMLs was not related to pain and that these features were reported alongside MRI‐detected features of OA (mainly joint space narrowing and osteophytes, which are poorly visualized in the small joints on x‐ray) and that this trend increased with age,^19^ in keeping with previous studies.^36^ In a second study we have published, when comparing a control group (with no foot pain) to a midfoot pain group and radiographic confirmed midfoot OA group, the size of the BMLs was associated with midfoot pain.^3^ This suggests the possible mechanism of foot orthoses to modify foot BMLs may be applied to stress‐related and OA‐related BMLs.

Using devices to modify or reduce bone stress has been explored to reduce injury and improve pain. Immobilization therapy is perhaps the most well‐known treatment for acute injuries of the foot and ankle, and walker boots or casts have been shown to reduce and resolve BMLs over one to four months.^20^, ^21^ Outside of this treatment, less in known about the biomechanical properties of in‐shoe devices and their influence on bone stress. An important experimental study of functional foot orthoses reported that an immediate reduction of bone stress (using an invasive bone‐mounted strain‐gauges) was evidenced in the tibial and second metatarsal bones upon initiation of orthotic use.^37^, ^38^ This is supported by a finite element imaging model of midfoot OA, showing navicular and cuneiform cartilage and bone stress was modified by changes in orthoses stiffness and arch height.^39^ The response of bone to external device has also been shown by wearing an in‐shoe pad. After running for two weeks with a pad placed on the lateral midfoot (aimed to induce foot pronation), baseline and follow‐up MRI scans showed BMLs occurred in lateral foot bones in 10 of 11 cases.^40^ More clinical studies have followed to test whether orthotic devices fitted to support the foot during running and intense training can reduce or prevent bone stress. In a military randomized controlled sham trial, in‐shoe foot orthoses reduced foot and lower leg injuries more than the sham group^23^ and, specifically, the incidence of medial tibial stress was lower.^41^ These studies support the gait studies, which suggest that foot orthoses can alter foot biomechanics,^42^ and it shows the potential of orthoses to support bone adaptation and prevent injury. In contrast, running shoes designed to simulate barefoot running and reduce support of the foot show increase in BMLs in the metatarsals.^43^ These studies show the potential for BMLs to be induced and modified in a relatively short period (from two to seven weeks).

Taking into account the previous research, this novel exploratory study suggests that wearing orthoses for 12 weeks can reduce midfoot pain and BML size compared to the control sham group. The orthoses intervention also reduced short‐term pain and impairment more than the control insole. The reductions in pain were similar to those reported in plantar fasciitis and posterior tibial tendon dysfunction randomized trials.^3^, ^4^, ^44^, ^45^, ^46^ These results in the foot also align with a knee brace study, which also showed knee pain and patellofemoral BML size were reduced in people with OA.^47^ This is important as longitudinal studies of the knee and foot suggest there is a relationship between the size of the BML, higher pain levels,^48^ and longer duration of pain,^32^ suggesting reducing pain related midfoot BMLs may have long‐term benefits and further studies are needed.

This exploratory study had limitations, especially the size of the study, that limited assumptions and generalizability, as a sample size calculation was not performed. Due to the small sample and number of midfoot bones, an unequal randomization method (2:1 ratio) was chosen, favoring greater numbers in the intervention group, as one of the main concerns was BML migration in the intervention group, which has been show in a one‐year follow‐up study.^49^ This therefore limited direct comparison between the intervention and control groups. The outcome measure to assess pain and impairment MMFPDI may have also limited the study, as both groups showed similar reductions over 12 weeks despite differences in overall patient‐reported improvement. This is in agreement with a footwear intervention study, in which the responsiveness of the MMFPDI was limited in an older population with foot pain.^50^

This study results may also be affected by response bias from both the researcher and participant, which may lead to greater positive results in the intervention group. To limit bias, the researcher presented each intervention device equally to the participants; however, patient beliefs were not fully assessed, and this would be recommended for future studies. The MRI results were analyzed anonymously without knowledge of baseline, follow‐up, and intervention group status to limit bias. Finally, this study set out to exclude people with radiographic OA, which may be less responsive to modification of BMLs over a short period. The baseline semiquantitative scoring showed MRI‐detected BMLs were detected alongside joint changes typical of OA,^19^ which may have affected the results.

In conclusion, foot orthoses are widely prescribed for foot pain, however the mechanism of action is not fully understood. To date, the physiologic effect of foot orthoses to mediate internal foot forces requires further research in clinical community populations, particularly in relation to bone stress. This exploratory study addresses some of this gap and provides data that shows that patient improvement, reduction in midfoot pain, and reduction in BML size were greater in the foot orthoses group than the control sham group.

## AUTHOR CONTRIBUTIONS

All authors contributed to at least one of the following manuscript preparation roles: conceptualization AND/OR methodology, software, investigation, formal analysis, data curation, visualization, and validation AND drafting or reviewing/editing the final draft. As corresponding author, Dr Halstead confirms that all authors have provided the final approval of the version to be published and takes responsibility for the affirmations regarding article submission (eg, not under consideration by another journal), the integrity of the data presented, and the statements regarding compliance with institutional review board/Declaration of Helsinki requirements.

## Supporting information


**Disclosure form**.

**Supplementary File 1:** Orthoses description and fitting

**Supplementary File 2:** Image Analysis ‐ Quantification of Bone Marrow Lesion Volume

**Supplementary File 3:** Flow chart of participants through the study (CONSORT 2010 statement)

**Data S1:** CONSORT 2010 checklist of information to include when reporting a randomised trial

## References

[1] Gates LS , Arden NK , Hannan MT , et al. Prevalence of foot pain across an international consortium of population‐based cohorts. Arthritis Care Res (Hoboken) 2019;71(5):661–670. 30592547 10.1002/acr.23829 PMC6483849

[2] Thomas MJ , Peat G , Rathod T , et al. The epidemiology of symptomatic midfoot osteoarthritis in community‐dwelling older adults: cross‐sectional findings from the Clinical Assessment Study of the Foot. Arthritis Res Ther 2015;17(1):178. 26166410 10.1186/s13075-015-0693-3 PMC4499901

[3] Arnold JB , Halstead J , Martín‐Hervás C , et al. Bone marrow lesions and magnetic resonance imaging‐detected structural abnormalities in patients with midfoot pain and osteoarthritis: a cross‐sectional study. Arthritis Care Res (Hoboken) 2023;75(5):1113–1122. 35593411 10.1002/acr.24955 PMC10952448

[4] Banwell HA , Mackintosh S , Thewlis D . Foot orthoses for adults with flexible pes planus: a systematic review. J Foot Ankle Res 2014;7(1):23. 24708560 10.1186/1757-1146-7-23 PMC4108129

[5] Mills K , Blanch P , Chapman AR , et al. Foot orthoses and gait: a systematic review and meta‐analysis of literature pertaining to potential mechanisms. Br J Sports Med 2010;44(14):1035–1046. 19996330 10.1136/bjsm.2009.066977

[6] Koushesh S , Shahtaheri SM , McWilliams DF , et al. The osteoarthritis bone score (OABS): a new histological scoring system for the characterisation of bone marrow lesions in osteoarthritis. Osteoarthritis Cartilage 2022;30(5):746–755. 35124198 10.1016/j.joca.2022.01.008 PMC9395274

[7] Mandell JC , Khurana B , Smith SE . Stress fractures of the foot and ankle, part 1: biomechanics of bone and principles of imaging and treatment. Skeletal Radiol 2017;46(8):1021–1029. 28374052 10.1007/s00256-017-2640-7

[8] Cowley E , Marsden J . The effects of prolonged running on foot posture: a repeated measures study of half marathon runners using the foot posture index and navicular height. J Foot Ankle Res 2013;6(1):20. 23705863 10.1186/1757-1146-6-20 PMC3668212

[9] Freund W , Weber F , Billich C , et al. The foot in multistage ultra‐marathon runners: experience in a cohort study of 22 participants of the Trans Europe Footrace Project with mobile MRI. BMJ Open 2012;2(3):e001118. 10.1136/bmjopen-2012-001118 PMC3364457 22619270

[10] Tenforde AS , Outerleys J , Bouxsein ML , et al. Metatarsal bone marrow edema on magnetic resonance imaging and its correlation to bone stress injuries in male collegiate basketball players. Orthop J Sports Med 2022;10(1):23259671211063505. 35071655 10.1177/23259671211063505 PMC8777350

[11] Greeves JP , Beck B , Nindl BC , et al. Current risks factors and emerging biomarkers for bone stress injuries in military personnel. J Sci Med Sport 2023;26(suppl 1):S14–S21. 37188615 10.1016/j.jsams.2023.04.006

[12] Miskovsky S , Khambete P , Faraji N , et al. Prevalence of asymptomatic talar bone marrow edema in professional ballet dancers: preliminary data from a 2‐year prospective study. Orthop J Sports Med 2023;11(5):23259671231159910. 37152549 10.1177/23259671231159910 PMC10159254

[13] Katakura M , Clark R , Lee JC , et al. Foot and ankle MRI findings in asymptomatic professional ballet dancers. Orthop J Sports Med 2024;12(8):23259671241263593. 39143984 10.1177/23259671241263593 PMC11322932

[14] Barr AJ , Campbell TM , Hopkinson D , et al. A systematic review of the relationship between subchondral bone features, pain and structural pathology in peripheral joint osteoarthritis. Arthritis Res Ther 2015;17(1):228. 26303219 10.1186/s13075-015-0735-x PMC4548899

[15] Zanetti M , Bruder E , Romero J , et al. Bone marrow edema pattern in osteoarthritic knees: correlation between MR imaging and histologic findings. Radiology 2000;215(3):835–840. 10831707 10.1148/radiology.215.3.r00jn05835

[16] Aso K , Sugimura N , Wada H , et al. Increased nerve growth factor expression and osteoclast density are associated with subchondral bone marrow lesions in osteoarthritic knees. Osteoarthr Cartil Open 2024;6(3):100504. 39176036 10.1016/j.ocarto.2024.100504 PMC11340585

[17] Hansen RT , Chenu C , Sofat N , et al. Bone marrow lesions: plugging the holes in our knowledge using animal models. Nat Rev Rheumatol 2023;19(7):429–445. 37225964 10.1038/s41584-023-00971-z

[18] Fang H , Zhang X , Wang J , et al. The relationship between MRI‐detected hip abnormalities and hip pain in hip osteoarthritis: a systematic review. Rheumatol Int 2024;44(10):1887–1896. 39136786 10.1007/s00296-024-05678-2 PMC11393093

[19] Halstead J , Martín‐Hervás C , Hensor EMA , et al. Association between clinical and MRI‐detected imaging findings for people with midfoot pain, a cross‐sectional study. J Foot Ankle Res 2025;18(1):e70019. 39797599 10.1002/jfa2.70019 PMC11724207

[20] Elias I , Zoga AC , Schweitzer ME , et al. A specific bone marrow edema around the foot and ankle following trauma and immobilization therapy: pattern description and potential clinical relevance. Foot Ankle Int 2007;28(4):463–471. 17475141 10.3113/FAI.2007.0463

[21] Aigner N , Radda C , Meizer R , et al. Bone marrow edema in the foot ‐ MRI findings after conservative therapy. Foot Ankle Surg 2005;11(2):87–91.

[22] Bonanno DR , Murley GS , Munteanu SE , et al. Effectiveness of foot orthoses for the prevention of lower limb overuse injuries in naval recruits: a randomised controlled trial. Br J Sports Med 2018;52(5):298–302. 29056595 10.1136/bjsports-2017-098273

[23] McCormick CJ , Bonanno DR , Landorf KB . The effect of customised and sham foot orthoses on plantar pressures. J Foot Ankle Res 2013;6(1):19. 23680496 10.1186/1757-1146-6-19 PMC3663766

[24] Chapman GJ , Halstead J , Redmond AC . Comparability of off the shelf foot orthoses in the redistribution of forces in midfoot osteoarthritis patients. Gait Posture 2016;49:235–240. 27459418 10.1016/j.gaitpost.2016.07.012 PMC5038933

[25] de Boer AG , van Lanschot JJ , Stalmeier PF , et al. Is a single‐item visual analogue scale as valid, reliable and responsive as multi‐item scales in measuring quality of life? Qual Life Res 2004;13(2):311–320. 15085903 10.1023/B:QURE.0000018499.64574.1f

[26] Averbuch M , Katzper M . Assessment of visual analog versus categorical scale for measurement of osteoarthritis pain. J Clin Pharmacol 2004;44(4):368–372. 15051743 10.1177/0091270004263995

[27] Pincus T , Bergman M , Sokka T , et al. Visual analog scales in formats other than a 10 centimeter horizontal line to assess pain and other clinical data. J Rheumatol 2008;35(8):1550–1558. 18597409

[28] Garrow AP , Papageorgiou AC , Silman AJ , et al. Development and validation of a questionnaire to assess disabling foot pain. Pain 2000;85(1–2):107–113. 10692609 10.1016/s0304-3959(99)00263-8

[29] Cook C , Cleland J , Pietrobon R , et al. Calibration of an item pool for assessing the disability associated with foot pain: an application of item response theory to the Manchester Foot Pain and Disability Index. Physiotherapy 2007;93(2):89–95.

[30] Kamper SJ , Maher CG , Mackay G . Global rating of change scales: a review of strengths and weaknesses and considerations for design. J Man Manip Ther 2009;17(3):163–170. 20046623 10.1179/jmt.2009.17.3.163 PMC2762832

[31] Tonbul M , Guzelant AY , Gonen A , et al. Relationship between the size of bone marrow edema of the talus and ankle pain. J Am Podiatr Med Assoc 2011;101(5):430–436. 21957275 10.7547/1010430

[32] Zanetti M , Steiner CL , Seifert B , et al. Clinical outcome of edema‐like bone marrow abnormalities of the foot. Radiology 2002;222(1):184–188. 11756724 10.1148/radiol.2221010316

[33] Thiryayi WA , Thiryayi SA , Freemont AJ . Histopathological perspective on bone marrow oedema, reactive bone change and haemorrhage. Eur J Radiol 2008;67(1):62–67. 18337044 10.1016/j.ejrad.2008.01.056

[34] Tarantino U , Greggi C , Cariati I , et al. Reviewing bone marrow edema in athletes: a difficult diagnostic and clinical approach. Medicina (Kaunas) 2021;57(11):1143. 34833361 10.3390/medicina57111143 PMC8625152

[35] González‐Martín D , Herrera‐Pérez M , Martín‐Vélez P , et al. Prevalence of bone marrow edema in a study population with foot and/or ankle pain. Foot (Edinb) 2019;40:76–80. 31136917 10.1016/j.foot.2019.04.004

[36] Zubler V , Mengiardi B , Pfirrmann CWA , et al. Bone marrow changes on STIR MR images of asymptomatic feet and ankles. Eur Radiol 2007;17(12):3066–3072. 17619194 10.1007/s00330-007-0691-1

[37] Meardon SA , Edwards B , Ward E , et al. Effects of custom and semi‐custom foot orthotics on second metatarsal bone strain during dynamic gait simulation. Foot Ankle Int 2009;30(10):998–1004. 19796595 10.3113/FAI.2009.0998

[38] Ekenman I , Milgrom C , Finestone A , et al. The role of biomechanical shoe orthoses in tibial stress fracture prevention. Am J Sports Med 2002;30(6):866–870. 12435654 10.1177/03635465020300061801

[39] Zhang H , Lin Lv M , Yang J , et al. Computational modelling of foot orthosis for midfoot arthritis: a Taguchi approach for design optimization. Acta Bioeng Biomech 2020;22(4):75–83. 34846024

[40] Schweitzer ME , White LM . Does altered biomechanics cause marrow edema? Radiology 1996;198(3):851–853. 8628882 10.1148/radiology.198.3.8628882

[41] Franklyn‐Miller A , Wilson C , Bilzon J , et al. Foot orthoses in the prevention of injury in initial military training: a randomized controlled trial. Am J Sports Med 2011;39(1):30–37. 21041512 10.1177/0363546510382852

[42] Jafarnezhadgero A , Esmaeili A , Hamed Mousavi S , et al. Effects of foot orthoses application during walking on lower limb joint angles and moments in adults with flat feet: a systematic review with meta‐analysis. J Biomech 2024;176:112345. 39353247 10.1016/j.jbiomech.2024.112345

[43] Ridge ST , Johnson AW , Mitchell UH , et al. Foot bone marrow edema after a 10‐wk transition to minimalist running shoes. Med Sci Sports Exerc 2013;45(7):1363–1368. 23439417 10.1249/MSS.0b013e3182874769

[44] Kulig K , Reischl SF , Pomrantz AB , et al. Nonsurgical management of posterior tibial tendon dysfunction with orthoses and resistive exercise: a randomized controlled trial. Phys Ther 2009;89(1):26–37. 19022863 10.2522/ptj.20070242

[45] Landorf KB , Keenan AM , Herbert RD . Effectiveness of foot orthoses to treat plantar fasciitis: a randomized trial. Arch Intern Med 2006;166(12):1305–1310. 16801514 10.1001/archinte.166.12.1305

[46] Baldassin V , Gomes CR , Beraldo PS . Effectiveness of prefabricated and customized foot orthoses made from low‐cost foam for noncomplicated plantar fasciitis: a randomized controlled trial. Arch Phys Med Rehabil 2009;90(4):701–706. 19345789 10.1016/j.apmr.2008.11.002

[47] Callaghan MJ , Parkes MJ , Hutchinson CE , et al. A randomised trial of a brace for patellofemoral osteoarthritis targeting knee pain and bone marrow lesions. Ann Rheum Dis 2015;74(6):1164–1170. 25596158 10.1136/annrheumdis-2014-206376 PMC4771926

[48] Zhang Y , Nevitt M , Niu J , et al. Fluctuation of knee pain and changes in bone marrow lesions, effusions, and synovitis on magnetic resonance imaging. Arthritis Rheum 2011;63(3):691–699. 21360498 10.1002/art.30148 PMC3056156

[49] Fernandez‐Canton G , Casado O , Capelastegui A , et al. Bone marrow edema syndrome of the foot: one year follow‐up with MR imaging. Skeletal Radiol 2003;32(5):273–278. 12679846 10.1007/s00256-003-0622-4

[50] Menz HB , Auhl M , Ristevski S , et al. Comparison of the responsiveness of the foot health status questionnaire and the Manchester foot pain and disability index in older people. Health Qual Life Outcomes 2014;12(1):158. 25344024 10.1186/s12955-014-0158-4 PMC4213529

