# Supplementary material for: Effect of Foot Orthoses on Midfoot Pain and the Volume of Bone Marrow Lesions in the Midfoot: A Randomized Mechanism of Action Study
Source: Arthritis Care Res (Hoboken). 2025 Dec 8;78(4):547–56. doi: 10.1002/acr.25648 (PMC13034095; doi:10.1002/acr.25648)
Supplement: Supplementary file 2 — Supplementary File 1: Orthoses description and fitting [file ACR-78-547-s001.docx]

**Supplementary File 1** **Orthoses description and fitting**

The intervention for this study was a pre-formed orthotic device called Vectorthotic**^®^** (Healthy Step Ltd, UK), see Figure 1a and 1b. Vectorthotic**^®^** are modifiable in-shoe foot orthoses, consisting of a composite polypropylene plastic shell with a heel cup and a contoured arch fitted to the foot and ending at the proximal region of the metatarsal heads. These are equipped with adjustable medial hindfoot posts that aim to support inversion of the hindfoot by 2, 4 or 6 degrees. The orthoses were finished by adding a full length 4mm compressed closed cell polyethylene foam cover with a brushed Nylon top sheet. In this study an optional Vectorthotic^®^ Extra cover with midfoot support was provided (increasing thickness from 4mm to 14mm in the midfoot) to optimise the potential functional effect of the device on the medial midfoot region by maximising arch contact.

Each participant who was randomised to the intervention arm received a pair of Vectorthotic**^®^** devices that was modified to maximise arch contact and support by either heat moulding, adding 4 or 6 degree medial wedge and or adding an Extra cover to ensure a close contact at the medial arch and produce a foot posture index score of zero or neutral posture. The forefoot additions included in the Vectorthotic**^®^** pack were not used in this study.

All participants were given verbal and written information regarding functional orthoses use and a daily diary to record maximum hours worn in footwear.

*Note The foot posture index is a 25 point continuum quantifying foot posture from -12 cavoid to 0 neutral and planus +12. Scored with the patient standing in relaxed standing position (Redmond et al. 2006 Clin Biomech 21(1):89-98).*

To provide a control comparative arm, participants were given a full-length cushioned top-cover insole that comprises of 4mm of compressed (closed cell polyethylene) foam with a brushed Nylon top sheet (Healthy Step Ltd, UK). This thin insole did not have any functional features associated with prescription orthotic devices (a rigid, contoured shell, a stabilising heel cup or applied wedges to position the foot joints) and was proposed as a control (sham) intervention.

All participants were given verbal and written information regarding functional orthoses use and a daily diary to record maximum hours worn in footwear

**Photo of the Vectorthotic^®^ pack:** *includes the orthotic shell, the posts and the top cover*

***
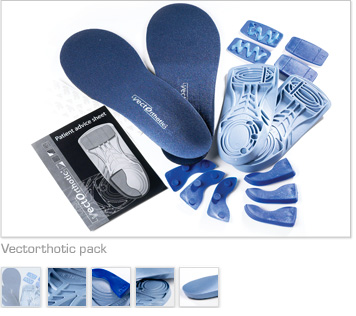
***
