# Supplementary material for: Effect of Foot Orthoses on Midfoot Pain and the Volume of Bone Marrow Lesions in the Midfoot: A Randomized Mechanism of Action Study
Source: Arthritis Care Res (Hoboken). 2025 Dec 8;78(4):547–56. doi: 10.1002/acr.25648 (PMC13034095; doi:10.1002/acr.25648)
Supplement: Supplementary file 3 — Supplementary File 2: Image Analysis ‐ Quantification of Bone Marrow Lesion Volume [file ACR-78-547-s004.docx]

**Supplementary File 2 Image Analysis - Quantification of Bone Marrow Lesion Volume**

The measurement of bones and BMLs were conducted using anonymised MRIs.

The BML was defined as the hyper-intense signal visualised on T2-weighted fat saturated sequences, this was measured using Analyze software, version 10 Analyze-Direct (Lenexa, Kansas USA), see Image 2a long axis T2-weighted sequence.

For each bone identified with BMLs, the bone was segmented in two-dimensions to create a three-dimensional render (see Image 2b bone volume render). The borders of bone were identified using a manual (spline) tracing function (using polynomial lines that semi-automates the tracing process) for each cross-sectional area per slice of bone (see 2c Analyze V10 software view).

To measure BML volume, signal was identified by manually identifying the lower and upper range of normal marrow greyscale signal (see Image 2d). Normal greyscale was subtracted from the bones through an automated function leaving the defined BML hyper-intense bone signal (see Image 2e).

Analyze software provided 2 outputs, a cross-sectional slice area of the bone and a cross-sectional area defined as BML signal. With the additional information of slice thickness and the slice gap, the volume of bone and BML per bone was calculated (Volume = ∑ Area Bone x (slice thickness + inter-slice gap).

| **2a Long axis T2 weight fat saturated sequence** | **2b Bone volume render** |
| --- | --- |
| **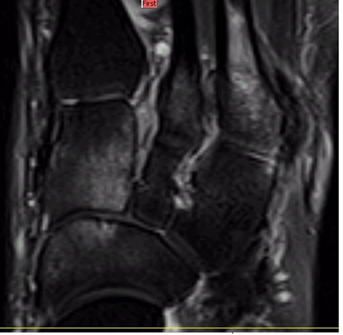** | **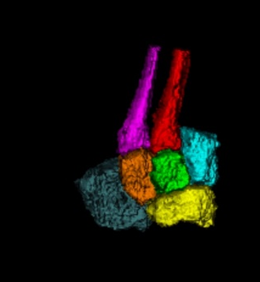** |

| **2c Analyze Version 10 segmentation view**  In primary plane in long axis | Secondary short axis plane |
| --- | --- |
| 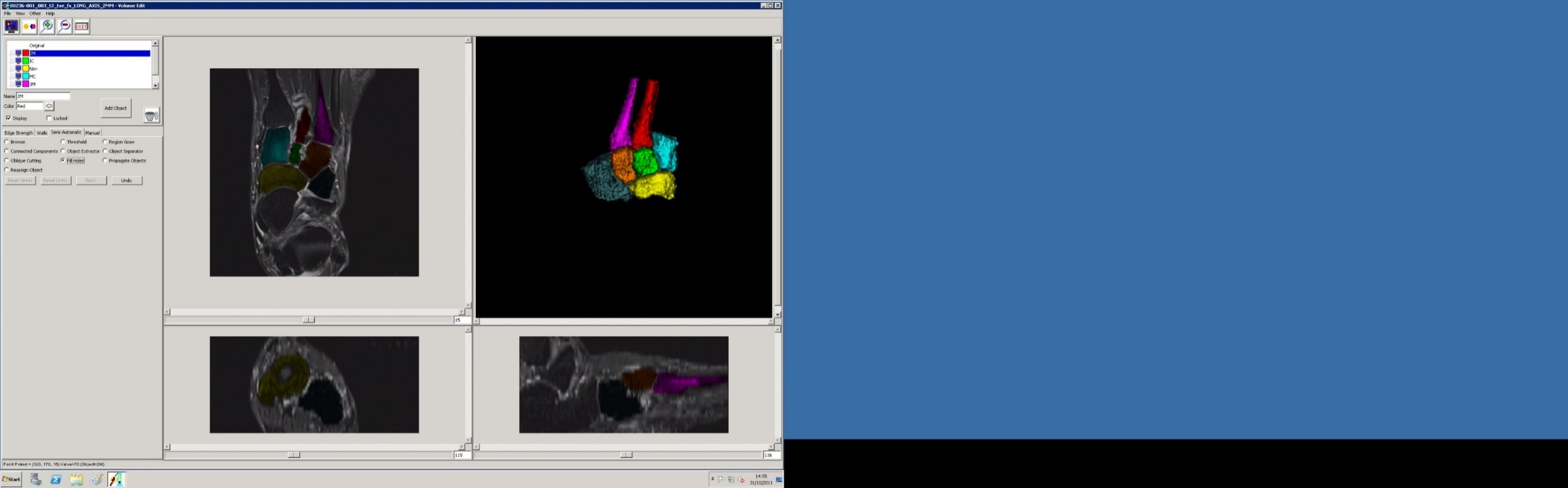 | 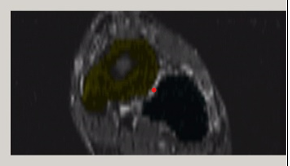 |
|  | Secondary sagittal plane |
|  | **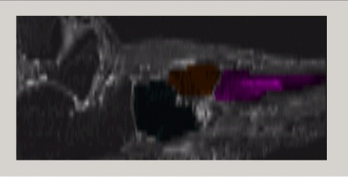** |

| **2d Analyze Version 10 segmentation** – to identify normal bone marrow signal |
| --- |
| 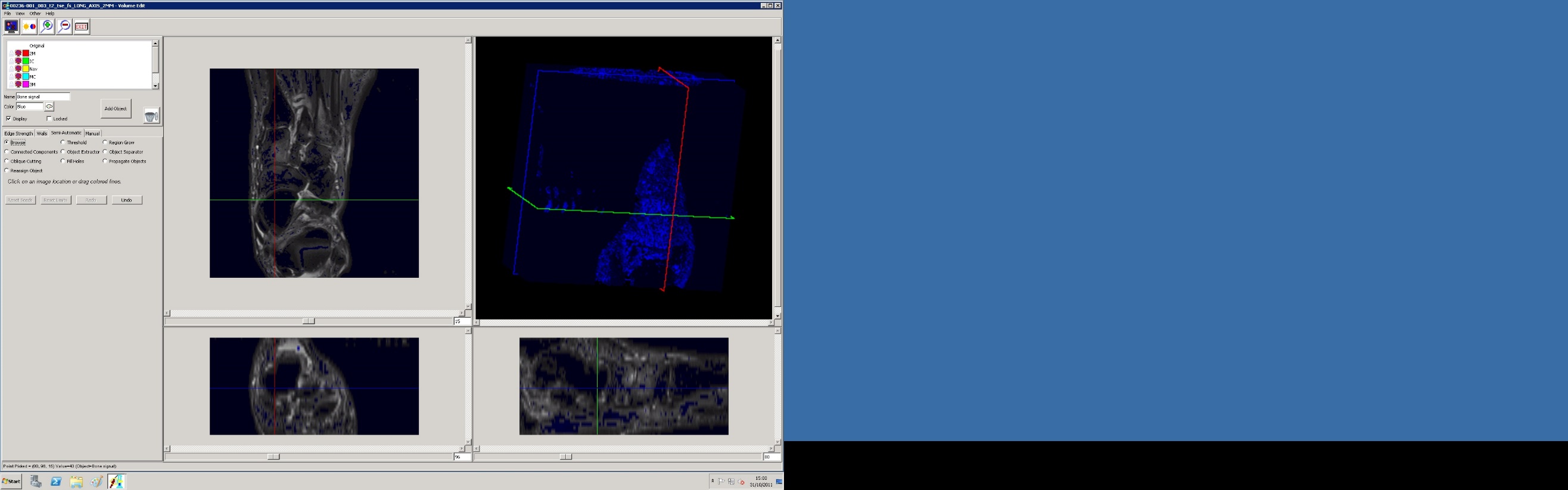 |

| **2e Analyze Version 10 Segementation -** Subtraction of grey scale from bone render |
| --- |
| 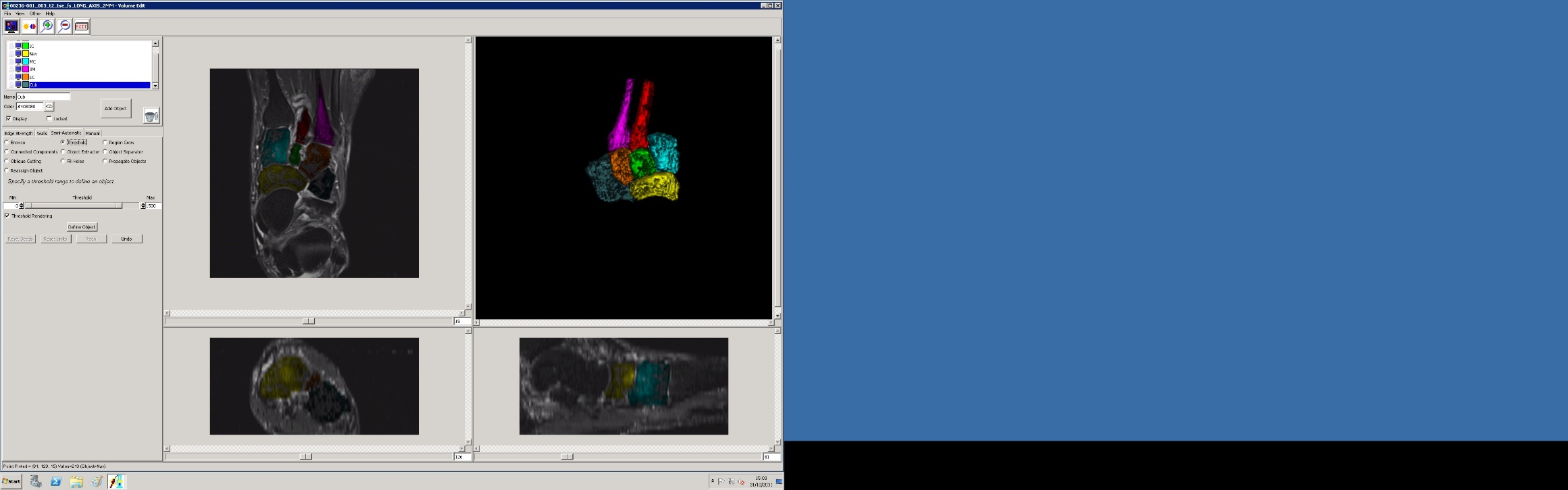 |
