# Supplementary material for: Effect of Foot Orthoses on Midfoot Pain and the Volume of Bone Marrow Lesions in the Midfoot: A Randomized Mechanism of Action Study
Source: Arthritis Care Res (Hoboken). 2025 Dec 8;78(4):547–56. doi: 10.1002/acr.25648 (PMC13034095; doi:10.1002/acr.25648)
Supplement: Supplementary file 4 — Supplementary File 3: Flow chart of participants through the study (CONSORT 2010 statement) [file ACR-78-547-s002.docx]

**Supplementary File 3**

**Flow chart of participants through the study (CONSORT 2010 statement)**

**________________________________________________________________________**

Assessed for eligibility (n=85)

MRI scan (n=61)

Enrolment

MRI evaluation BMLs identified

(n=45 invited)

**3 declined study after randomisation orthoses group**

Randomisation

(n=45)

Randomisation

(n=42)

Allocations

Orthoses (n=27)

Foot orthoses

Control (n=15)

Cushioning Insole

Follow-up

1^st^ Follow-up (n=25)

6 weeks follow-up

2 Excluded due to fall

1^st^ Follow-up (n=15)

6 weeks follow-up

2^nd^ Follow-up (n=22)

12 weeks follow-up

3 Excluded
- 1 Fall

- 1 Flare of back pain

- 1 lost to follow-up

2^nd^ Follow-up (n=15)

12 weeks follow-up

Analysis (n=15)

Analysis (n=22)

Analysis
